# A two-stage strategy for methanogenesis suppression and rapid acetogenic biofilm formation in microbial electrosynthesis

**DOI:** 10.3389/fmicb.2025.1655259

**Published:** 2025-10-31

**Authors:** Jacopo Ferretti, Marika A. J. Zegers, Marco Zeppilli, Ludovic Jourdin

**Affiliations:** ^1^Department of Chemistry, University of Rome Sapienza, Rome, Italy; ^2^Department of Biotechnology, Delft University of Technology, Delft, Netherlands; ^3^e-Refinery Institute, Delft University of Technology, Delft, Netherlands

**Keywords:** microbial electrosynthesis, CO_2_ reduction, biofilm, mixed culture, chain elongation, mixotrophy, methanogenesis

## Abstract

The practical implementation of microbial electrosynthesis (MES) is currently limited by the slow microbial colonisation of the electrode and the need to suppress methanogenic activity. This study investigates a two-stage strategy to suppress methanogenesis and promote the rapid formation of an acetogenic biofilm in a directed-flow-through bioelectrochemical reactor. Four start-up regimes were compared: mixotrophic without heat pre-treatment (M), mixotrophic with heat pre-treatment (MT), heterotrophic without heat pre-treatment (H), and heterotrophic with heat pre-treatment (HT), each followed by a common autotrophic phase. Mixotrophy outperformed heterotrophy by accelerating and increasing acetate accumulation. However, adding heat pre-treatment (MT) introduced a short lag phase and resulted in less sustained chain elongation than mixotrophy alone (M). Under the mixotrophic regime, microbial analysis showed an enrichment of genera with acetogenic representatives such as *Clostridium sensu stricto 12* and *Sporomusa*, alongside a reduction in facultative anaerobic and fermentative bacteria. Full biofilm colonisation of the electrode was achieved within 55 to 65 days, while acetate, butyrate, and caproate production was initiated within the first week, reaching concentrations typically observed only after approximately 70 days under autotrophic conditions. Methane remained undetectable for about 40 days and, when detected later, exhibited low coulombic efficiencies (< 1%). Taken together, these results indicate that mixotrophic start-up provides a promising route to accelerate electrode colonisation and enhance early-stage productivity in MES, while highlighting the need for further optimisation and a deeper understanding of microbial interactions.

## 1 Introduction

In recent years, microbial electrosynthesis (MES) has gained considerable interest as an innovative method for reducing CO_2_ emissions through conversion into higher value products. In MES, microorganisms interact with a solid-state cathode as an electron donor to drive reductive metabolism. The resulting products include short-chain carboxylic acids, alcohols and methane (Zeppilli et al., 2020b; Dessì et al., 2021a; Cabau-Peinado et al., 2024). The production of carboxylic acids in MES is bio-catalysed by acetogenic bacteria. Although significant advancements have been made in MES in recent years, the production rates and coulombic efficiencies of short-chain carboxylic acids and alcohols remain limited by reactions that compete for CO_2_ and e^−^/H_2_, notably hydrogenotrophic methanogenesis, and by product loss through acetoclastic methanogenesis (Deppenmeier and Müller, 2008; Schlegel and Müller, 2013). This is because methanogenic species have a thermodynamic advantage over acetogens, especially at low H_2_ concentration (Ragsdale and Pierce, 2008; Borrel et al., 2016). Given the competitive advantage of methanogens and their detrimental impact on MES performance, mitigating methanogenic activity has become a critical area of focus.

Recent studies have examined both abiotic and biotic strategies to suppress methanogenesis, as its prevention is critical to minimise electron losses to methane. A common approach is to use methanogen-specific inhibitors like 2-bromoethanesulfonate (BESA). However, a metagenomic study observed that the addition of BESA to the system does not necessarily guarantee the inhibition of methanogens, as sulphate reducing bacteria have been demonstrated to consume BESA (Scholten et al., 2000; Bian et al., 2021; Mills et al., 2022). The inhibitory effect of BESA can also disappear over time, as methanogens can adapt with repeated dosing of BESA. Additionally, the use of BESA is cost-prohibitive at industrial scale (Jourdin et al., 2020). Operational conditions have also been adjusted to disfavour methanogens. In one study, modifying the cathode with conductive magnetite nanoparticles steered electrons towards acetogenesis, increasing acetate yields by up to 8.5-fold while reducing methane output (Viggi et al., 2020). Biotic strategies, such as selective enrichment or tailored feeding regimes, also aimed to promote homoacetogenic and chain-elongating bacteria instead of methanogens (Mills et al., 2022). However, no single strategy has completely prevented methane production in MES.

Heat-shock treatment has also been used to selectively reduce methanogenic populations. Previous experiments have shown that thermal treatment of the inoculum partially inhibits methanogens (Zeppilli et al., 2020a, 2022; Cristiani et al., 2022). This treatment aimed to select for acetogens by exploiting their spore-forming capacity and greater heat tolerance, thereby promoting their enrichment (Levinson and Hyatt, 1970; Bayliss et al., 1981; Akhtar et al., 2009; Mills et al., 2022). While heat-shock effectively knocks out methanogens, it also reduces populations of key acetogens (e.g., *Acetobacterium*), potentially limiting the chain length of carboxylates produced (Mills et al., 2022). This underscores the need for more selective and sustainable methanogenesis control methods that can reliably inhibit methanogenic activity without impairing carboxylate production.

The autotrophic metabolism of acetogens, using the Wood Ljungdahl pathway (WLP), requires the investment of 1 mol of ATP, which is recovered by phosphorylation at the substrate level with the production of 1 mol of acetate. To generate additional ATP, acetogens rely on ATP synthase driven by an H^+^ or Na^+^ gradient. on the presence of cytochromes, they produce approximately 0.3 to 0.5 mol of ATP per mol of acetate, posing an energetic constraint that makes growth challenging (Schuchmann and Müller, 2014; Kremp et al., 2022). To overcome this limitation, acetogens can exploit a mixotrophic metabolism (concurrent use of organic and inorganic substrates). The WLP is well-suited to mixotrophy because it requires less ATP than other CO_2_-fixation pathways and can couple NAD(P)H generated during glycolysis together with reduced ferredoxin to reduce two mol CO_2_ to one mol acetyl-CoA (Fast and Papoutsakis, 2012; Tracy et al., 2012; Jones et al., 2016). It has been observed that growth on lactate and glucose in the presence of syngas increased acetate production, with acetate as the major product in non-model acetogens such as *Blautia producta* and *Thermoanaerobacter kivui*. When glycerol was fed instead of lactate, more reduced acids than acetate, such as butyrate, valerate, caproate and alcohols were produced, mostly under mixotrophic conditions (Maru et al., 2018). Similar observations were made in a study where model acetogens such as *Clostridium acetobutylicum, Clostridium ljungdahlii, Clostridium autoethanogenum, Moorella thermoacetica*, and *Eubacterium limosum* were tested (Jones et al., 2016). In mixotrophy studies with *Acetobacterium woodii*, an increase in biomass production was also observed in the co-presence of H_2_/CO_2_ and organic carbon (Braun and Gottschalk, 1981; Sandoval-Espinola et al., 2017). Furthermore, mixotrophy has been used for *Clostridium beijerinckii* to improve the conversion of carbon into biobutanol, which would otherwise result in a two-third loss as CO_2_ under heterotrophic fermentation conditions (Sandoval-Espinola et al., 2017).

Mixotrophic growth of acetogens in MES can also be used as a strategy to overcome the limitations of strictly autotrophic operation. The metabolic flexibility of acetogens in mixotrophy improves energy conservation and stimulates faster metabolism, leading to shorter lag phases and higher growth rates (O'Keeffe et al., 2024). For example, co-feeding pentose sugars with syngas eliminated the lag phase and enabled immediate growth of *Clostridium autoethanogenum*, reaching cell densities about three-fold higher than in autotrophic cultures (Oppelt et al., 2024). Similarly, studies have demonstrated that mixotrophic conditions can lead to higher biomass yields and product titers in MES, as the simultaneous utilisation of CO_2_ and organic substrates provides a more robust energy supply for cellular processes (Jones et al., 2016; Emerson et al., 2019; Park et al., 2019; Mann et al., 2020; Chu et al., 2022). However, these studies primarily used suspended-cell systems and did not assess electrode colonisation, leaving the effects of mixotrophy on biofilm formation and microbial selection at the electrode surface insufficiently explored.

This study aimed to evaluate strategies for simultaneously suppressing methanogenesis and accelerating acetogenic biofilm formation in MES. While previous studies have often addressed these challenges separately, their combined optimisation remains underexplored, particularly in biofilm-driven MES systems, where early electrode colonisation is critical. To this end, a series of inoculum pre-treatments and operational regimes were tested in directed-flow-through bioelectrochemical (DFBR) reactors (Cabau-Peinado et al., 2024). As a pre-treatment, a thermal shock was applied at the start of the experiment to reduce methanogen viability and give acetogens a competitive advantage. Following this pre-treatment, MES was sequentially carried out under two operational stages: (1) a mixotrophic regime and (2) an autotrophic regime. Although methanogens can utilise hydrogen and acetate present in the reactor, they lack the metabolic flexibility of acetogens and cannot exploit mixotrophic metabolism, which gives acetogens a thermodynamic and competitive advantage under these conditions. This metabolic advantage could facilitate the rapid formation of an acetogenic biofilm on the electrode surface, while washing out the methanogens. To understand the effect of organic carbon on the production and formation of an acetogenic biofilm, Stage 1 was also tested under heterotrophic regime, where N_2_ was flushed into the system instead of CO_2_. This was done to remove CO_2_ produced during fermentation to avoid its use by methanogens (Scholten et al., 2000; Bian et al., 2021; Mills et al., 2022). By following this approach, a deeper insight into the interaction between carbon sources, microbial competition and reactor performance in MES could be gained.

## 2 Materials and methods

### 2.1 MES reactor configuration

**Figure 1:**
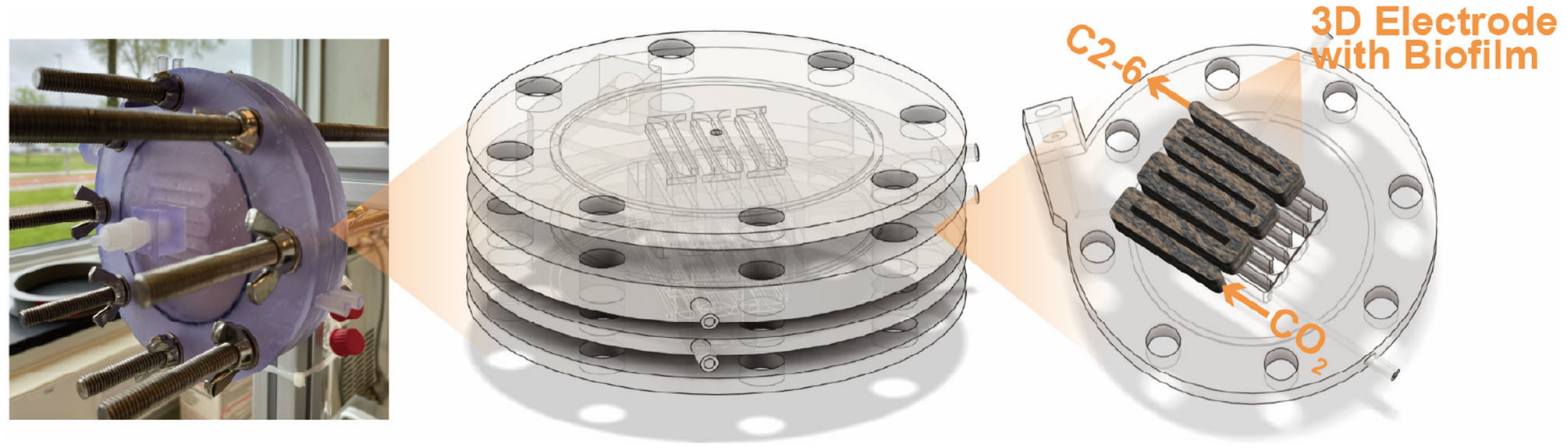
Complete reactor stack consisting of a 3D carbon felt on the cathode side and a Pt/IrO_2_ coated 2D titanium electrode on the anode side. The stack is enclosed by two supporting back-plates, which each have a titanium rod coming through that connects the electrodes to the potentiostat. The entire stack is sealed using an NBR O-rings.

Four bioelectrochemical reactors were assembled. The serpentine DFBR proposed by Cabau-Peinado et al. was adapted, with the new configuration provided in Figure 1 (Cabau-Peinado et al., 2024). The design was changed from square to circular to improve sealing and reduce the risk of leakage.

Each reactor consisted of a cathodic and anodic compartment of the same volume (6.3 mL), enclosed between supporting back plates that held the current collectors in place and provided stability to the stack. The serpentine flow path in the anode matched that of the cathode to ensure identical hydrodynamics in both compartments (see Supplementary Information 1 for detailed schematics and dimensions). The back plates included depressions to accommodate the electrodes and ensure consistent positioning within the serpentine channels (Supplementary Information 1). The individual components of the reactors were 3D printed using the Form 3B printer (Formlabs, USA) with the biocompatible BioMed Clear Resin V1 (Formlabs, USA). The anode consisted of a titanium plate coated with Pt/IrO_2_ (Insoluble Anode Technology (IAT) B.V., Netherlands), while the cathode electrode material was unmodified carbon felt (CTG Carbon GmbH, Germany). Before being assembled into the reactor, the carbon felt underwent treatment with 1 M HCl (1 day), 1 M NaOH (1 day), and ozone gas as described in previous work (Winkelhorst et al., 2023). The total volume of the carbon felt in the cathodic chamber was 6.3 cm^3^ with a thickness of 0.5 cm. An isostatic graphite plate (GP) (3.2 mm thick, Fuel Cell Store, USA) was used as a current collector onto which a conductive coating (graphite conductive adhesive, Electron Microscopy Sciences, USA) was applied to the interface with the carbon felt to improve the electrical connection between both materials. The compartments were separated by a cation exchange membrane (CEM) with a projected surface area (PSA) of 12.5 cm^2^ (CMI-7000s, Membrane International Inc.). Sealing between all reactor plates was ensured using rubber NBR O-rings (82.27 × 1.78 mm) placed at multiple points along the assembly (Supplementary Information 1).

The catholyte pH was monitored using a pH probe (QP108X, ProSense, Netherlands) installed in the recirculation loop of the catholyte outside the reactor and controlled at pH 5.8 with an AQUIS touch S (Jumo) controller. The catholyte recirculation loop was also equipped with a bubble column, which was used to flush solely N_2_ or a mixture of CO_2_ and N_2_ (50% v/v each). The total volume of the catholyte, which included the cathodic chamber, the bubble column and all tubing in the loop was 0.09 L. The anolyte was maintained at pH 2 and its pH was checked regularly during operation. During the operation, the reactors were maintained at 30 °C by placing them in a temperature-controlled chamber.

### 2.2 MES reactor operation

The medium composition contained 0.2 g L^−1^ NH_4_Cl, 0.015 g L^−1^ CaCl_2_·2H_2_O, 0.04 g L^−1^ MgCl_2_·6H_2_O, 8.1 g L^−1^ KH_2_PO_4_, 0.9 g L^−1^ Na_2_HPO_4_, and 1 mL L^−1^ of a trace element solution. The trace element solution contained 1.5 g L^−1^ FeCl_3_·6H_2_O, 0.15 g L^−1^ H_3_BO_3_, 0.03 g L^−1^ CuSO_4_·5H_2_O, 0.18 g L^−1^ KI, 0.12 g L^−1^ MnCl_2_·4H_2_O, 0.06 g L^−1^ Na_2_MoO_4_·2H_2_O, 0.12 g L^−1^ ZnSO_4_·7H_2_O, 0.15 g L^−1^ CoCl_2_·6H_2_O, 0.023 g L^−1^ NiCl_2_·6H_2_O, and 10 g L^−1^ EDTA. In both the heterotrophic and mixotrophic conditions in Stage 1, 3.5 g L^−1^ glucose, 2 g L^−1^ fructose and 2 g L^−1^ Gibco™ Bacto™ Peptone were added. The composition of the anolyte was identical to the catholyte medium used at that specific time, without the trace element solution, sugars and peptones. The pH of the anolyte was also lowered to 2 with phosphoric acid (H_3_PO_4_) to facilitate proton transfer across the membrane.

The reactors were operated in continuous mode with a hydraulic retention time (HRT) of 4 days, calculated from the total catholyte volume (0.09 L). Both the catholyte and anolyte were continuously circulated at a flow rate of 30 mL min^−1^. While the heterotrophic reactors were supplied with only N_2_ during Stage 1, dissolved CO_2_ was supplied by continuously sparging a CO_2_:N_2_ 50:50 gas mixture at a rate of 0.1 L min^−1^ into the cathodic bubble column in the mixotrophic reactors during Stage 1 and in all reactors during Stage 2. The reactors were connected in a three-electrode configuration to a multichannel potentiostat (VSP-300, BioLogic, France). When polarised, the reactors were run at −63 mA, which corresponds to −5.04 mA ${\text{cm}}_{\text{PSA}}^{-2}$, and cathode potential time series for each reactor are provided in Supplementary Information 2. Constant current was chosen to impose the same electron flux across reactors. A 3M Ag/AgCl reference electrode (QM710X, ProSense, Netherlands) was used in each reactor and positioned directly adjacent to the outlet of the serpentine channel in the cathodic compartment (Supplementary Information 3).

Each reactor was inoculated on day 0 with 0.05 g_COD_ L^−1^ of an enriched mixed microbial culture, consisting of a community prepared by mixing the final cathodic biofilms from reactors described in earlier work (Winkelhorst et al., 2023; Cabau-Peinado et al., 2024). A preselected community was chosen because the source reactors achieved high productivities of acetate, butyrate, and caproate. The inoculum was either used without treatment or was heat-treated at 120 °C for 2 h in an oven (Cristiani et al., 2022). The operation was divided into two stages for each reactor, as depicted in Table 1. Stage 1 started with a short 5-day batch for each reactor before starting continuous mode.

**Table 1:** Overview of experimental reactor conditions (mixotrophic or heterotrophic) and inoculum treatments (heat-treated or not).

**Reactor name**	**Heat treatment inoculum**	**Conditions stage 1 (day 0–30)**	**Conditions stage 2 (day 30–85)**
M	No	Mixotrophic: CO_2_ + electrical current + sugars/peptones	Autotrophic: CO_2_/N_2_ + electrical current
MT	Yes		
H	No	Heterotrophic: N_2_ + sugars/peptones	
HT	Yes		

### 2.3 Analytical methods

Liquid samples (3 mL) were collected from each reactor twice a week. The optical density of unfiltered samples was measured at 600 nm with a UV-VIS spectrophotometer (UV-1800 series, Shimadzu, Japan) to quantify planktonic cells. Samples were filtered using a 0.2 μm microporous filter, and their total nitrogen content was analysed using a TOC analyser coupled with a TN unit (TOC-L series total organic carbon analysers, Shimadzu, Japan). The furnace temperature was maintained at 720 °C.

The concentration of C2 to C6 carboxylic acids and alcohols were analysed using a gas chromatographer (Thermofisher, USA) with a Stabil-wax™ column of 25 m length and 0.2 μm internal diameter. The column temperature was maintained at 50 °C for 7 min, increased to 180 °C for 8 min, and held at that temperature for 9 min. Helium was used as the carrier gas at a flow rate of 1 mL min^−1^, and the flame ionisation detector was maintained at 250 °C. Gas samples were taken from the bubble column of the cathode loop and injected into a CompactGC4.0 (Global Analyser Solutions, The Netherlands). The GC was equipped with a TCD detector with He as carrier gas and had a column temperature of 70 °C. Helium was used as the carrier gas at a flow rate of 1 mL min^−1^ and the ionisation detector was kept at 250 °C. In the available CompactGC4.0 configuration, methane was the only gas quantified, other gases were not monitored. The method detection limit for methane was 5 ppm. Biomass growth rates and specific production rates were calculated as described by Winkelhorst et al. (2023). In this case, carbon selectivity represents the fraction of carbon assimilated into a specific product relative to the total amount of carbon assimilated into all identified products, i.e., acetate, butyrate, caproate, and biomass. All mass and electron balances were performed as described in Supplementary Information 4.

### 2.4 DNA extraction and microbial community analysis

For all four operated MES reactors, DNA extraction was performed on the following samples: (1) the inoculum (day 0), (2) the bulk catholyte after the switch from mixotrophic/heterotrophic conditions to autotrophic (day 32) and (3) the biofilm at the end of the experiments (day 85). The DNA extraction was performed using DNeasy PowerSoil Pro Kit (Qiagen, Hilden, Germany), using all the corresponding solutions and equipment as specified by the manufacturer. The purified DNA underwent quantification using a Qubit broad-range assay [Qubit 2.0 Fluorometer and Qubit dsDNA BR Assay Kit (ThermoFischer Scientific)].

Microbial community analysis was conducted by 16S rRNA gene amplicon sequencing (Novogene Co., Ltd., Cambridge, UK). Amplicons were generated with primers 341F and 806R targeting V3–V4, and sequenced on an Illumina platform (2 x 250 bp). Reads were processed using the provider's pipeline. As 341F/806R has bacterial-centred coverage and does not comprehensively capture archaeal diversity, methanogens may be under-represented.

## 3 Results and discussion

This study examined whether methanogenic activity could be suppressed in mixed-culture, biofilm-driven MES without relying on costly chemical inhibitors, and whether electrode colonisation could be accelerated, two key challenges for the industrial application of MES. To test this, four reactors were operated across two stages, differing in carbon regime and inoculum treatment. The first stage used either heterotrophic (H and HT) or mixotrophic conditions (M and MT), followed by a second autotrophic MES stage. In each regime, one reactor was inoculated with heat-treated biomass (HT and MT) and the other with untreated biomass (H and M).

### 3.1 Microbial characterisation

**Figure 2:**
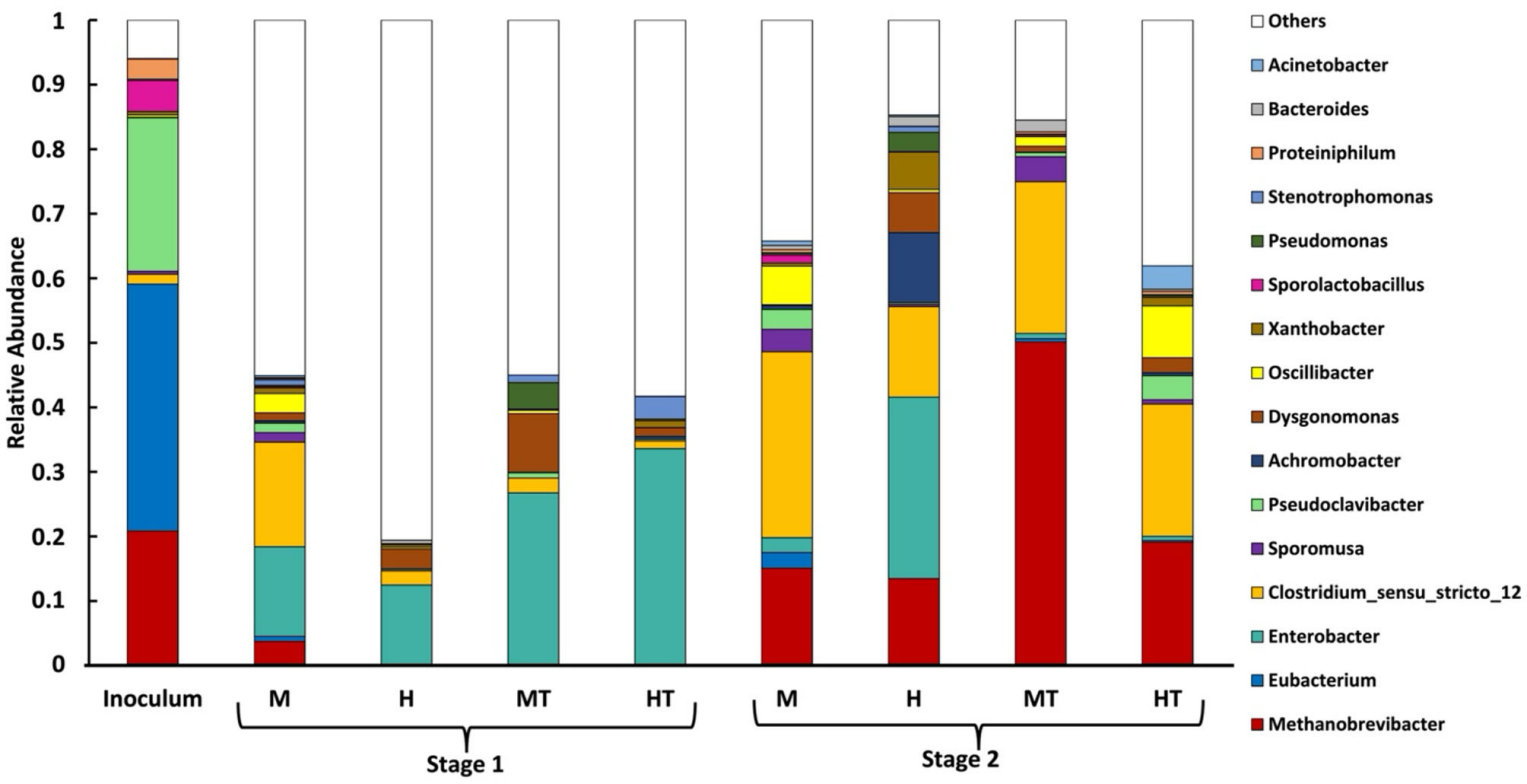
Relative abundance (% of total classified reads at genus level) of microbial genera in the planktonic phase during Stage 1 (day 0–30) and in the biofilm during Stage 2 (day 30–85) for reactors M, HT, MT, and H, alongside the initial inoculum. Plotted taxa are the top 16 genera by mean relative abundance across the samples in this study. “Others” comprises all remaining genera not included in the top 16 (each with mean relative abundance <0.05% across the samples in this study).

To understand the colonisation dynamics between acetogens and methanogens under different operational conditions, a microbial analysis at genus level was conducted. Planktonic samples were collected 2 days after the switch to Stage 2 (day 32), which corresponds to ~0.5 HRT. Consequently, residual DNA from non-viable cells may have persisted. As a substantial fraction of biomass is expected to attach to the cathode, as discussed in Section 3.7, planktonic profiles provide only a partial view of the community. Biofilm samples were taken directly from the electrode upon reactor disassembly on day 85. The inoculum was dominated by *Eubacterium*, followed by *Pseudoclavibacter, Methanobrevibacter, Sporolactobacillus*, and *Clostridium sensu stricto 12*, indicating the presence of both genera with acetogenic representatives and methanogenic populations prior to reactor inoculation (Figure 2).

#### 3.1.1 Stage 1 promotes biofilm adhesion by genera with acetogenic representatives and limits methanogens

In the Stage 1 planktonic samples (Figure 2), the abundance of the genus *Eubacterium* was considerably lower than in the inoculum (~0), suggesting that it either adhered to the electrode surface or was outcompeted in the suspended phase (Jourdin et al., 2015; Dessì et al., 2021b). During Stage 1, *Clostridium sensu stricto 12'*s relative abundance increased in M, whereas MT showed a weaker and delayed enrichment relative to M and closer to H/HT. Other genera, such as *Sporomusa* (a genus with acetogenic representatives) and *Oscillibacter* (fermentative, associated with chain elongation) were found in the planktonic phase. In contrast, the HT reactor exhibited a higher relative abundance of *Enterobacter*, a genus composed mainly of fermentative bacteria. In reactor H, the low abundance of *Methanobrevibacter* during Stage 1 likely reflects the heterotrophic start-up conditions, operation at pH 5.8, and dominance of fast-growing fermenters in the planktonic phase, all of which are unfavourable for hydrogenotrophic methanogens.

The heat treatment appeared to play an important role in inhibiting the growth of potentially competing bacteria under heterotrophic conditions. In reactor H, the fraction of reads unclassified to a genus during Stage 1 was highest among reactors, consistent with a more heterogeneous planktonic community in the absence of thermal selection. Furthermore, the presence of the genus *Methanobrevibacter* was detected in the M reactor but not in the heat-treated MT reactor, which is consistent with an inhibitory effect of heat treatment on methanogens. However, the relative abundance of *Clostridium sensu stricto 12* was higher in the M reactor than in MT, suggesting that the heat treatment may have also affected non-methanogenic populations.

#### 3.1.2 Methanogens persist in Stage 2 biofilm regardless of inoculum heat treatment

As observed across all reactors, the methanogen *Methanobrevibacter*, which was initially present in the inoculum, was detected in the biofilm on the final day. Its relative abundance ranged between 12% and 20%, although a higher value of ~50% was recorded in the MT reactor. To explain the persistence of methanogens within the biofilm, several possible mechanisms were considered based on the observed results. First, *Methanobrevibacter* may have been present in the biofilm from early on and thus not detectable in the Stage 1 planktonic profiles. Methanogens could preferentially occupy outer biofilm layers where access to residual H_2_ from the cathode is greater, exploiting their higher growth rates and strong H_2_ affinity (Pavlostathis et al., 1990). Second, the operating pH (5.8) may have been unfavourable for methanogenesis. However, prior work with the same inoculum at pH 5.8 under galvanostatic control (with BESA) still reported ~20% *Methanobrevibacter* at the end of the experiment (Cabau-Peinado et al., 2024), and methane production was observed in a miniaturised DFBR at pH 5.4 (Zegers et al., 2025). These findings indicate that pH 5.8 alone does not suppress methanogenesis and suggest that the chosen start-up and operating conditions may inhibit methanogenic activity rather than wash methanogens out of the biofilm and reactor.

#### 3.1.3 Stage 2 biofilms show higher relative abundance of *Clostridium sensu stricto 12* and *Sporomusa*

During Stage 2, the biofilm community became more homogeneous and showed higher relative abundances of *Clostridium sensu stricto 12, Sporomusa*, and *Oscillibacter*, alongside a decrease in the fermentative genus *Enterobacter*, relative to the Stage 1 planktonic community. The mixotrophic reactors (M, MT) showed a higher relative abundance of *Sporomusa* than the heterotrophic reactors (H, HT), suggesting that mixotrophy enriched a genus predominantly composed of acetogenic species, several of which are known to produce acetate from CO_2_ (Aryal et al., 2017). In the HT reactor, the highest relative abundance of *Oscillibacter* was observed. This genus has been reported to play a role in the bioelectrochemical production of butyrate (Dessì et al., 2021b). *Oscillibacter* was not detected in a previous study using the same inoculum and similar experimental conditions, but without the initial Stage 1 feeding phase (Cabau-Peinado et al., 2024). This suggests that *Oscillibacter* may require a period of growth on organic substrates before becoming established. It is also worth noting that the genus *Eubacterium* was only observed in the biofilm of reactor M, despite being dominant in the inoculum. Although *Eubacterium* is capable of glucose metabolism, its limited presence may reflect competitive dynamics rather than metabolic limitations, with reactor M potentially providing more favourable conditions for its establishment (Litty and Müller, 2021). *Clostridium* species are known to thrive under sugar-rich, anaerobic conditions due to their rapid growth and structured biofilm-forming capacity (Rendueles and Ghigo, 2015). Similarly, *Sporomusa ovata* has been shown to readily colonise cathodes and perform microbial electrosynthesis under mixotrophic conditions, which may explain the early establishment of the *Sporomusa* genus and its later prevalence in Stage 2 biofilms (Aryal et al., 2017).

#### 3.1.4 Mixotrophy as a key to controlling undesirable bacteria

In reactor H, a high relative abundance of *Enterobacter* was maintained (~27%). In addition, the relative abundance of genera with acetogenic activity (*Clostridium sensu stricto 12, Sporomusa, Eubacterium)* was lower than in the other reactors (< 15%), and the microbial community appeared more heterogeneous at steady state. Reactor H also displayed a high relative abundance of aerobic and facultative anaerobic genera such as *Achromobacter, Dysgonomonas, Xanthobacter*, and *Pseudomonas* (~24%), which are likely to have proliferated during Stage 1, as no thermal shock was applied to inhibit them, unlike in reactor HT. In the mixotrophic reactors M and MT, by contrast, a negligible amount of the aforementioned facultative anaerobic bacteria was observed during Stage 2, unlike in reactors H and HT, regardless of thermal treatment. This pattern may indicate that mixotrophy, during start-up, provides extra reducing power and readily usable organics that could help acetogens attach to the cathode. Once organics were removed, the established acetogenic biofilm likely continued to direct electrons and CO_2_ into products, leaving little room for facultative fermenters.

### 3.2 (Co-)feeding sugars/peptones promotes faster growth and quicker electrode colonisation

**Figure 3:**
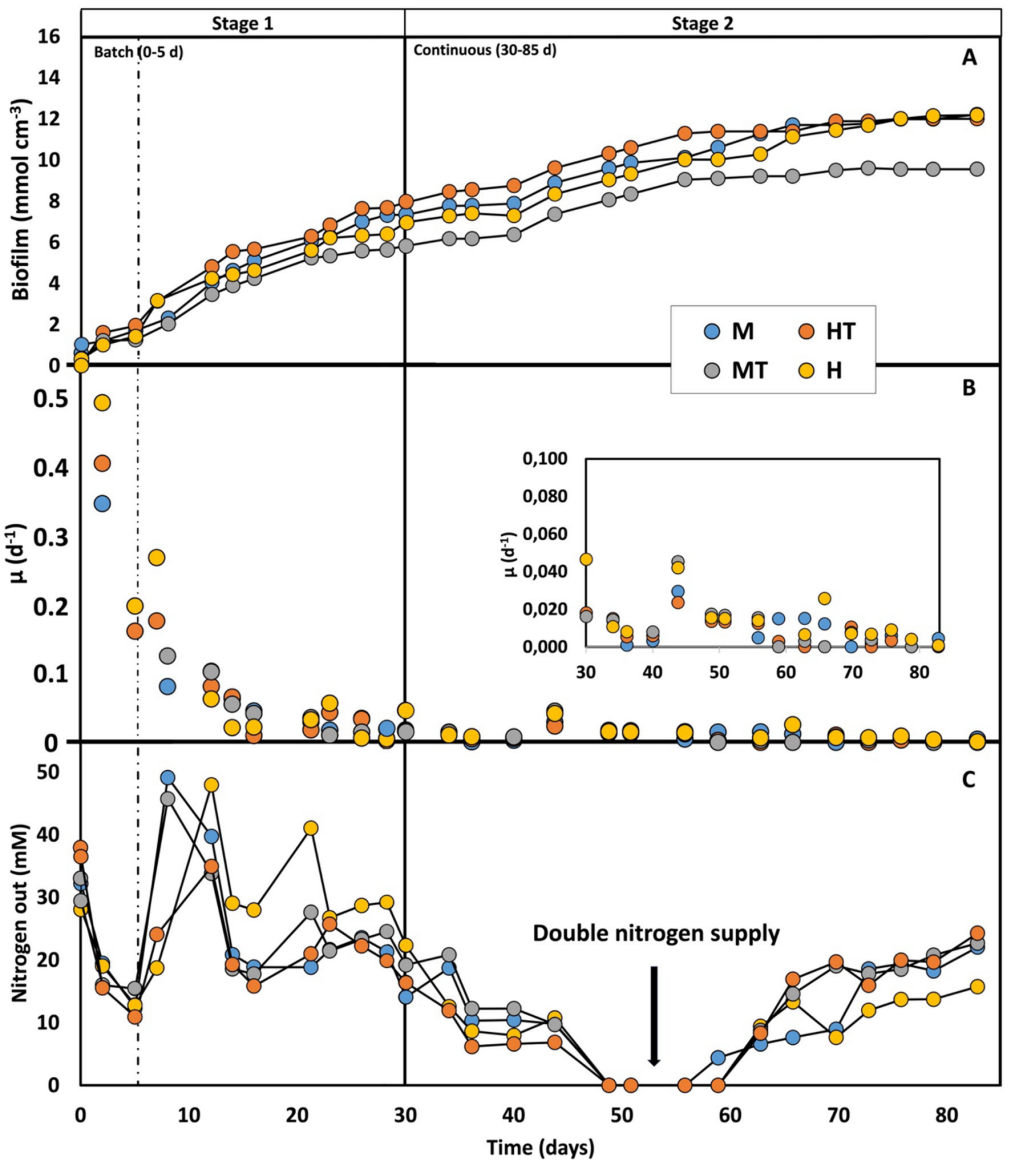
Biofilm concentration production **(A)**, biomass-specific uptake rate **(B)**, and nitrogen consumption **(C)** over time for the four operated reactors (M, MT, H, HT). The first dashed line indicates the end of the batch period and the beginning of the continuous mode (Stage 1); the second solid line indicates the beginning of the autotrophic phase (Stage 2).

**Figure 4:**
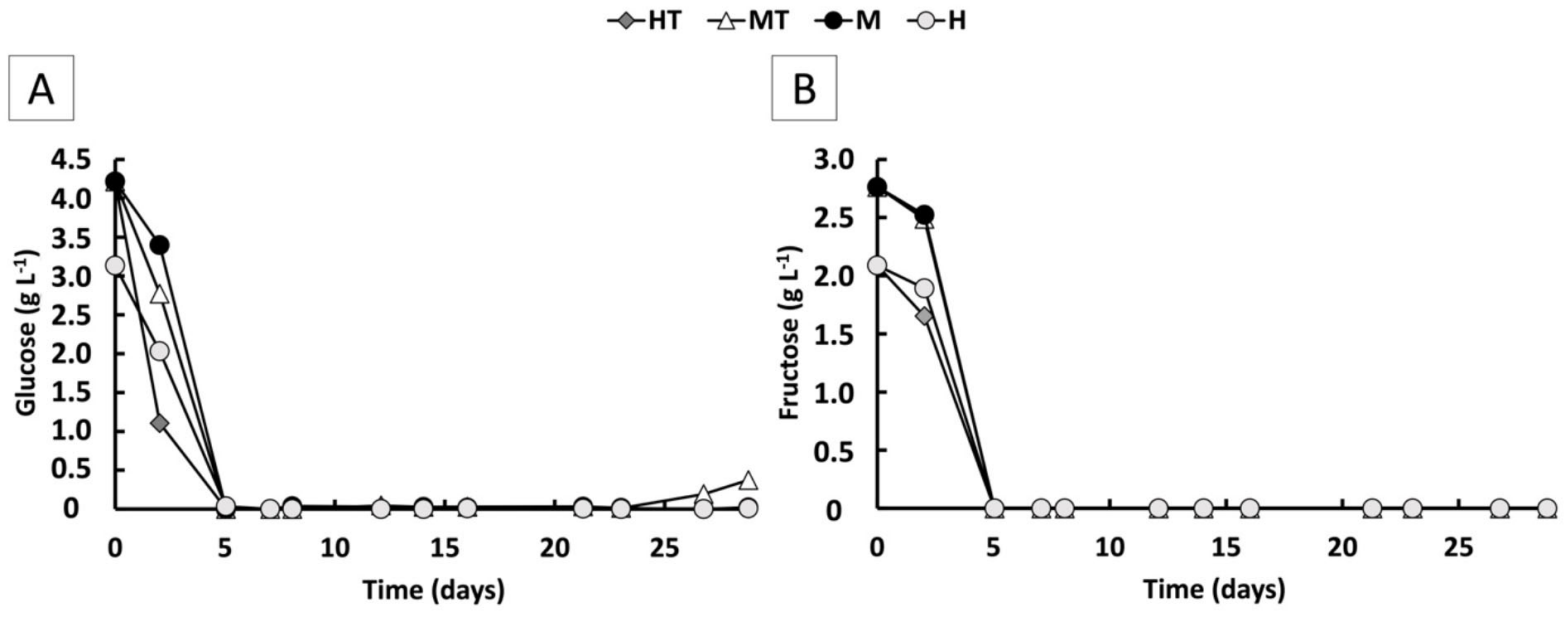
Glucose **(A)** and fructose **(B)** concentration over time for the four operated MES reactors (M, H, MT and HT).

During the first 5 days of the experiment, when the reactors were operated in batch mode, relatively high specific growth rates (μ) were observed, reaching a maximum of 0.5 d^−1^ (Figure 3B). This aligns with the availability of relatively high concentrations of fructose, glucose, and peptones, which likely supported rapid biomass growth. Both glucose and fructose were simultaneously consumed and fully depleted by day 5 in all four reactors (Figure 4). Based on the 5-day co-consumption period, the average consumption rates were 0.8 g L^−1^ d^−1^ for glucose and 0.5 g L^−1^ d^−1^ for fructose across all reactors. However, these values likely underestimate the actual rates, as glucose and fructose may have been depleted before day 5. Faster consumptions were observed in the first 2 days with the heat-treated inoculum (MT and HT) than with the non-treated biomass (M and H).

Once continuous mode was initiated in Stage 1, the μ declined to around 0.01 d^−1^. Despite this decrease, glucose and fructose consumption rates remained comparable to the batch phase (0.88 g L^−1^ d^−1^ for glucose and 0.50 g L^−1^ d^−1^), while reactor concentrations stayed near zero, indicating a metabolic preference for these substrates. Because continuous flow dilutes non-attached cells, glucose and fructose were likely used to support attachment and biofilm formation on the carbon felt (Figures 3A, C). Additionally, no clear differences in growth kinetics were observed between the different reactor conditions. In Stage 2, growth kinetics were again similar across all reactors, with μ's ranging between 0.005 and 0.02 d^−1^. These μ's also fall within the range reported in previous kinetic studies conducted under comparable experimental conditions (Winkelhorst et al., 2023; Cabau-Peinado et al., 2024). The most frequently observed μ in these works was approximately 0.02 d^−1^, occurring just before the plateau in biofilm accumulation per unit electrode volume (Figure 3A).

As shown in Figure 3A, the amount of biofilm on the electrode reached a plateau between days 55 and 65, which can be interpreted as full colonisation of the electrode (approximately 12.20 mmol cm^−3^) (Winkelhorst et al., 2023; Cabau-Peinado et al., 2024). The maximum theoretical amount of dry biomass per electrode volume was calculated to be around 12 mmol cm^−3^, based on a cell density of 1.09 g cm^−3^, a dry weight ratio of 30%, 5 and a carbon felt porosity of 90% (Cabau-Peinado et al., 2024). This supports the conclusion that the observed plateau corresponds to full biofilm colonisation of the electrode. Reactor MT reached a slightly lower amount of biomass (9.55 mmol cm^−3^), possibly due to heterogeneity in the packing of the cathode material (Zegers et al., 2025). A decrease in the specific growth rate over time was also observed, similar to the trend reported by Winkelhorst et al. (2023). However, direct comparison is limited due to differences in feeding strategies. It is worth noting that the μ observed in Stage 2 of this study closely resemble those reported by Cabau-Peinado et al. with similar reactor design, inoculum source, and operating conditions, but using a purely autotrophic feed and a slightly larger volume of carbon felt (9.5 cm^3^ vs. 6.24 cm^3^). In that study, full electrode colonisation occurred only after 225 days under both galvanostatic and potentiostatic conditions (Cabau-Peinado et al., 2024). In contrast, the use of a mixotrophic/heterotrophic regime during the first stage in this study led to complete colonisation within just 55–65 days, despite a comparable inoculum concentration. These findings demonstrate that (co-)feeding sugars and peptones during the start-up phase of a two-stage process promotes faster growth and accelerates electrode colonisation.

### 3.3 The coexistence of electric current and organic substrates enhances the start-up phase of MES (Stage 1)

**Figure 5:**
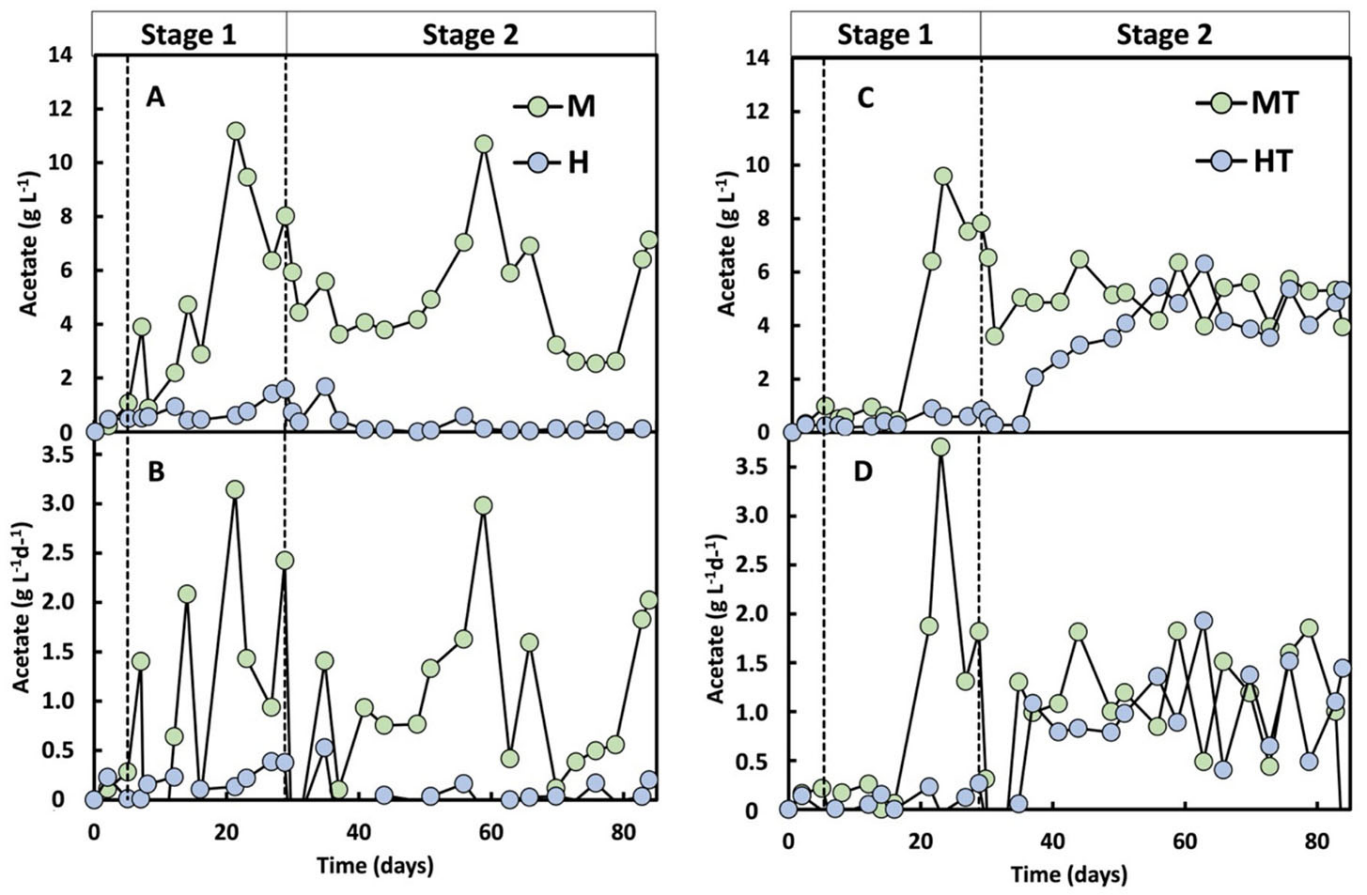
Acetate concentration **(A, C)** and acetate production rate **(B, D)** over time for the four operated MES reactors (M, H, MT and HT). The first dashed line indicates the end of the batch period and the beginning of the continuous mode (Stage 1); the second dashed line indicates the beginning of the autotrophic phase (Stage 2).

**Figure 6:**
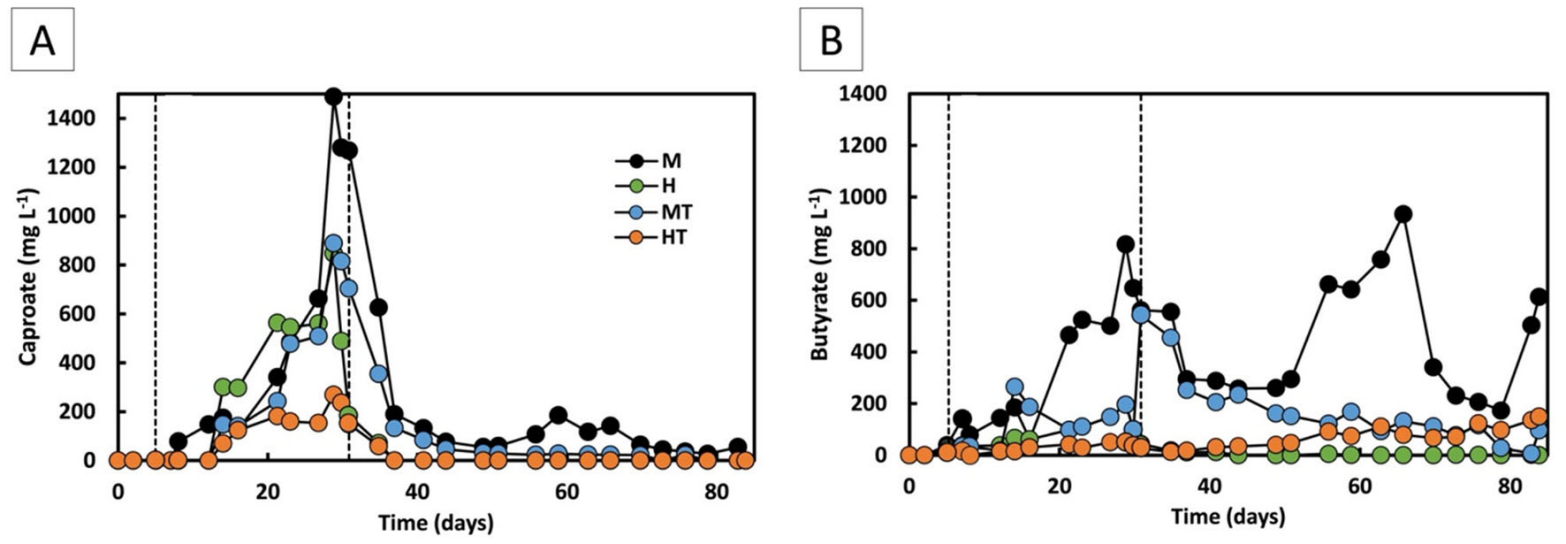
Caproate **(A)** and butyrate **(B)** concentrations over time for the four operated MES reactors (M, H, MT, and HT). The first dashed line indicates the end of the batch period and the beginning of the continuous mode (Stage 1); the second dashed line indicates the beginning of the autotrophic phase (Stage 2).

Stage 1 began with a short 5-day batch phase before switching to continuous mode. During the first 5 days, acetate reached 3.9 g L^−1^ in M and 1.0 g L^−1^ and MT, vs. 0.5 g L^−1^ in H and 0.5 g L^−1^ in HT, suggesting rapid activation of mixotrophic metabolism (Figures 5A, C). During continuous operation, acetate increased earlier and to higher concentrations in the mixotrophic reactors (M, MT) than in the heterotrophic reactors (H, HT). From day 21 to the end of Stage 1, a 28% decline in acetate concentration was observed in reactor M, coinciding with the onset of butyrate (0.8 g L^−1^) and caproate (1.5 g L^−1^) production (Figure 6). Acetate production rates in the heterotrophic reactor H were lower but more consistent throughout Stage 1 (Figure 5B), with concentrations peaking towards the end of Stage 1 at 1.6 g L^−1^ (Figure 5A). Butyrate and caproate were also produced in reactor H, up to 0.05 and 0.6 g L^−1^, respectively (Figure 6).

In the reactors inoculated with heat-treated biomass, a distinct difference in performance was observed. Despite the rapid activation seen during the batch phase in the MT reactor, an ~8-day lag phase followed the start of continuous feeding, an effect commonly reported after thermal shocks (Cristiani et al., 2022; Zeppilli et al., 2022). Consistent with the earlier community analysis (Section 3.1), Stage 1 profiles showed a lower relative abundance of the *Clostridium sensu stricto 12* genus in MT than in M, indicating that heat treatment likely affected non-methanogenic populations. From day 21 onward, the acetate production rate in MT increased to 1.8 g L^−1^ d^−1^, reaching a peak production rate of 3.7 g L^−1^ d^−1^ and concentration of 9.6 g L^−1^ by the end of Stage 1 (Figures 5C, D). Reactor HT reached a significantly lower maximum acetate concentration of 0.8 g L^−1^. Similar to reactor M, MT showed a decline in acetate production from day 26 onwards, accompanied by butyrate and caproate production from day 14 (Figure 6). The HT reactor, however, produced low amounts of butyrate and maintained lower acetate production rates (0.12 g L^−1^ d^−1^) throughout Stage 1. Caproate did reach a concentration of approximately 0.3 g L^−1^ by the end of Stage 1.

These Stage 1 results indicate that the mixotrophy significantly improved the start-up phase of acetate production. Acetate concentrations of 11.2 g L^−1^ in reactor M and 9.5 g L^−1^ in reactor MT were reached by days 21 and 23, respectively. By contrast, a previous study using the same inoculum under autotrophic MES conditions in a similar galvanostatic reactor required approximately 110 days to reach comparable concentrations (Cabau-Peinado et al., 2024). The close alignment in reactor design and inoculum origin enables a meaningful comparison between the two datasets. Increased acetate production under mixotrophic conditions is attributed to redox balancing, where electron carriers reduced during substrate oxidation are re-oxidised via the WLP, thereby increasing CO_2_ conversion rates (Schuchmann and Müller, 2016). Furthermore, studies on *Acetobacterium woodii* have shown that certain organic substrates, such as glycine betaine, are degraded via pathways that consume CO_2_ and enhance acetyl-CoA and acetate formation (Lechtenfeld et al., 2018). Although glycine betaine was not supplied in the researched reactors, the presence of complex organics like peptones may have supported similar mixotrophic metabolism, involving CO_2_ fixation during the degradation of amino acids or related compounds. This could explain the higher acetate production observed under mixotrophic conditions in this study. A mixotrophic start-up also increases electron-donor availability (via the supplied organics), which may contribute to the faster onset of acetate formation alongside the redox-balancing effects described above.

A start-up phase with glucose supplementation has also been applied in other bioelectrochemical systems, including for hydrogen production from wastewater, where one of the highest reported hydrogen production rates (3.7 L d^−1^) was achieved (Baeza et al., 2017). In a separate study, a heterotrophic start-up phase was tested using 0.36 g L^−1^ glucose and the methanogenesis inhibitor 2-BESA (Zaybak et al., 2013). Current consumption began only after 2 weeks, and efficient reactor start-up required higher glucose concentrations (3.50 g L^−1^) and a cathode potential of −0.8 to −1.0 V vs. SHE. Additionally, other studies have demonstrated that feeding acetate instead of bicarbonate during start-up accelerate biocathode colonisation and improve hydrogen production rates (Jeremiasse et al., 2012; Zaybak et al., 2013; Baeza et al., 2017). These findings support the effectiveness of mixotrophic start-up phases. However, unlike in previous studies, the current work demonstrates that high acetate production can be rapidly achieved in enriched mixed-culture MES using peptone-rich medium and glucose/fructose supplementation, without external inhibitors or pre-treated inocula. This suggests that combining mixotrophy with a fixed current can provide a robust, scalable strategy for accelerating MES start-up and enhancing early-stage productivity.

### 3.4 MES enables stable acetate production from the onset of feeding regime shift (Stage 2)

From day 30 onwards, all reactors were switched to autotrophic conditions, with the organic carbon supply stopped. Under these conditions, all systems were operated identically, relying solely on CO_2_ sparging and an applied current (−50 A m^−2^, −10.1 kA m^−3^) as carbon and electron sources. Following the transition, reactor M exhibited a temporary drop in acetate production before stabilising over the following weeks and, towards the end of the experiment, increasing again to rates comparable to Stage 1 (Figure 5A). The MT reactor had a lag phase of only 1 day after the feeding regime switch, producing 5.1 g L^−1^ of acetate with a production rate of 1.1 g L^−1^ d^−1^, showing constant production for over 50 days (Figures 5C, D).

Reactor HT had a longer lag phase and began to produce acetate 6 days after the start of stage 2, with a relatively constant rate of 0.9 g L^−1^ d^−1^ until day 50, accumulating acetate up to 5 g L^−1^ (Figures 5C, D). After this period, acetate production reached a stable concentration of 4.7 g L^−1^, with a production rate of 1.1 g L^−1^ d^−1^. Even though HT was not acclimated for electrosynthesis in Stage 1 due to the lack of polarisation and CO_2_, the autotrophic production of acetate suggests that the biofilm produced under heterotrophic conditions consisted, at least partly, out of acetogens. A similar trend might have been expected in reactor H. However, it showed no carboxylate production: average production rates were close to zero, with variability caused by sporadic, low-level production peaks (Figure 5B). This lack of autotrophic activity suggests that the heterotrophy in Stage 1 was insufficient to support MES activity once organic carbon was removed. As shown in a meta-analysis of cathodic microbial communities, biofilms derived from inocula not subjected to heat shock contain a lower abundance of acetogens compared to those from heat-treated inocula (Mills et al., 2022). Figure 2 supports this interpretation, as reactor H had a more heterogeneous biofilm with *Enterobacter*, lower representation of genera with acetogenic species, and elevated aerobic/facultative genera (*Achromobacter, Dysgonomonas, Xanthobacter, Pseudomonas*). Reactor HT, by contrast, had a more prominent presence of *Clostridium sensu stricto 12* and *Sporomusa*.

### 3.5 Mixotrophy produces more reduced products than heterotrophy

During Stage 1, the mixotrophic reactors (M and MT) produced higher amounts of more reduced compounds than acetate, compared to the heterotrophic reactors (H and HT), in line with findings reported in the literature (Jones et al., 2016; Maru et al., 2018). In reactor M, butyrate production was higher and increased gradually over time compared to reactor H, reaching 0.82 g L^−1^ and close to 0 g L^−1^, respectively, by the end of Stage 1 (Figure 6B). This can be related back to the Stage 1 planktonic profiles, where M showed a higher relative abundance of *Clostridium sensu stricto 12*, a genus with acetogenic activity, whereas H/HT retained more fermentative genera such as *Enterobacter* (Figure 2). The butyrate production rate in reactor M remained around 0.06 g L^−1^ d^−1^ throughout most of Stage 1, rising to 0.15 g L^−1^ d^−1^ on the final day, whereas in reactor H it remained close to zero. A similar pattern was observed for caproate (Figure 6A). Reactor M reached a final concentration of 1.49 g L^−1^, compared to 0.89 g L^−1^ in reactor H. The production rate in reactor M increased from around 0.04 g L^−1^ d^−1^ to 0.4 g L^−1^ d^−1^ on the last day, while reactor H maintained a steady rate of about 0.03 g L^−1^ d^−1^ throughout Stage 1. Stage 1 showed more *Oscillibacter* in M than H, consistent with potential for reverse β-oxidation (Figure 2). In the reactors inoculated with heat-treated biomass, reactor MT also outperformed reactor HT in secondary acid production. Final butyrate concentrations were close to zero in HT and 0.6 g L^−1^ in MT. For caproate, MT produced four times more than HT, reaching 0.8 g L^−1^ vs. 0.2 g L^−1^. Consistently, Stage 1 profiles showed HT with more *Enterobacter* and less *Clostridium sensu stricto 12* than MT, aligning with its weaker secondary-acid formation (Figure 2).

The results from Stage 1 suggest that chain elongation pathway was more expressed in mixotrophic reactors than in heterotrophic reactors. Mixotrophy has been shown to enhance carbon conversion due to a greater availability of reducing power compared to both heterotrophy and autotrophy (Jones et al., 2016). Although the observed production of butyrate and caproate is consistent with microbial chain elongation, the contribution of fermentative pathways, especially under the sugar and peptone rich conditions applied, cannot be excluded (Kleerebezem and van Loosdrecht, 2007). Chain elongation occurs only under specific environmental conditions, including the presence of energy-rich compounds, reducing equivalents, and acetyl-CoA. Ethanol and lactate are commonly identified as electron donors in this process, as their oxidation to acetate makes reverse β-oxidation reactions thermodynamically favourable (Raes et al., 2017, 2020; Reddy et al., 2018). In the heterotrophic reactors (H and HT), chain elongation could have been supported by ethanol or lactate produced during the fermentation of glucose and fructose, alongside the availability of acetyl-CoA. While ethanol was not detected in any of the four reactors, this does not rule out its production and immediate consumption. However, the accumulation of excess ethanol would be more thermodynamically favourable (González-Cabaleiro et al., 2013). In contrast, the mixotrophic tests (M and MT) exhibited greater production, likely due to the increased generation of both NADH in the methyl branch of the WLP and acetyl-CoA from the reduction of CO_2_. Unlike fatty acid biosynthesis, where acetyl-CoA is first converted to the elongation unit malonyl-CoA at the cost of an ATP, reverse β-oxidation directly uses acetyl-CoA as the elongation unit (Wu et al., 2017; Garces Daza et al., 2023). An overproduction of substrates for this reaction would have likely induced the process already in the first operational days.

In Stage 2, a decrease in both the concentration and production rates of butyrate and caproate was observed in all reactors. No secondary acid production was detected in reactor H. Reactors MT and HT exhibited lower, irregular butyrate production and no detectable caproate for most of Stage 2. The main secondary acid produced in reactor M was butyrate, reaching 0.9 g L^−1^ by day 65 (Figure 6B). Caproate production in reactor M was limited, occurring primarily between days 48 and 58, with concentrations ranging from 0.05 to 0.19 g L^−1^ (Figure 6A). This transient production in reactor M suggests the presence of chain-elongating organisms, although their activity appeared to decrease over time. This decline coincided with the Stage 2 shift to more homogeneous biofilms enriched in *Clostridium sensu stricto 12* and *Sporomusa* (Figure 2), suggesting substrate limitation rather than absence of acetogenic and chain-elongating genera. The different trends between the mixotrophic reactors likely reflect the prior heat treatment in MT, which may have reduced chain-elongating genera during start-up, explaining why butyrate remained stable in M but declined in MT after ~day 50 (Figure 6B).

These findings on chain-elongation products align with previous work showing that chain elongation can occur under strict autotrophic conditions, although typically with longer operational times. For instance, stable production of butyrate and caproate was observed from around day 80 under similar autotrophic conditions, with production rates of approximately 0.25 g L^−1^ d^−1^ for both carboxylic acids reported at that time point (Cabau-Peinado et al., 2024). In earlier studies, production began only after day 150, at which point similar butyrate and caproate production rates were reported (Jourdin et al., 2018). This suggests that while the two-stage process supports early onset of chain elongation, a more prolonged acclimatisation period under autotrophic conditions may be necessary to achieve sustained or higher caproate production rates. Early butyrate onset under strict autotrophy has been reported in MES using alternating flow-through, with acetate appearing within 10 days, n-butyrate by day 13, and caproate by day 45 (de Smit et al., 2023).

### 3.6 Acetogenesis is favoured to the detriment of methanogenesis

**Figure 7:**
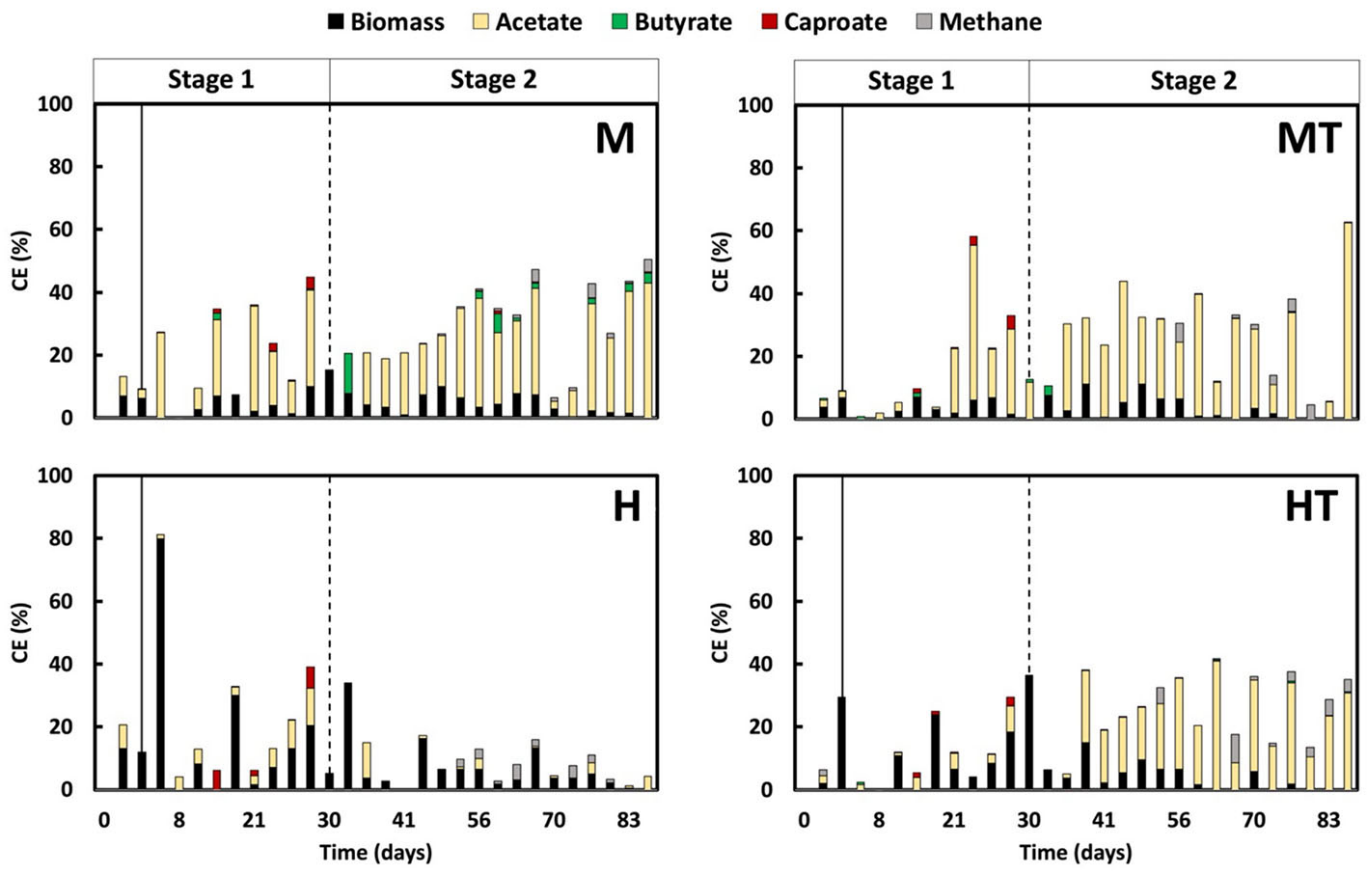
Coulombic efficiency (CE) into acetate, methane, biomass, butyrate (C4) and caproate (C6) over time in all reactors (M, MT, H, HT). The solid line indicates the end of the batch period and the beginning of the continuous mode (Stage 1); the second dashed line indicates the beginning of the autotrophic phase (Stage 2).

In all reactors, a similar trend in methanogenic activity was observed, with no methane being produced during Stage 1 (Figure 7). Even after switching to autotrophic MES conditions, methane remained undetectable until around day 40 in all cases regardless of whether the inoculum had undergone thermal shock. This suggests that the combination of thermal pre-treatment and a mixo- or heterotrophic start-up regime effectively delayed methanogen activity during early electrode colonisation. After day 40, methane CE increased slightly in all reactors but remained low (< 10%) throughout the experiment.

Among the reactors, MT and M showed the most effective suppression. Methane CE in MT peaked briefly at 5.7% on day 50, but dropped to below 1% the next day, and remained low for the rest of the experiment. In reactor M, methane CE fluctuated but did not exceed 4.0%, with values generally between 0.1% and 1.0%. As methane CE remained low (< 10%) throughout the experiment, variations in acetate CE could be attributed to concurrent formation of chain-elongation products, diversion of electrons to abiotic H_2_ evolution under the applied current, time-varying biomass synthesis and maintenance during colonisation, and mass-transfer and microenvironmental constraints (CO_2_ supply, pH).

The heterotrophic reactors displayed more variable outcomes. In reactor H, methane CE rose modestly between days 40 and 50 (max. 2.4%) before returning below 1%, while acetate CE remained low in Stage 1 and did not increase under autotrophic conditions. By comparison, reactor HT showed a continuous upward trend in methane CE, reaching 4.0% by day 82, the only reactor with such a progressive increase. This indicates that the thermal shock alone was insufficient to maintain long-term suppression in the absence of CO_2_, possibly due to faster recovery of methanogens or limited acetogenic competitiveness in the heterotrophic regime.

In previously reported studies, methane production typically began within the first 10–20 days or persisted despite the use of inhibitors such as BESA (Viggi et al., 2020). For instance, Zeppilli et al. (2022) observed methane formation within approximately 15–20 days following thermal pretreatment and Cristiani et al. (2022) reported methanogenesis onset around day 20 despite initial suppression (Cristiani et al., 2022; Zeppilli et al., 2022). The extended period of methanogenesis suppression observed in the current work, with no detectable methane for up to 40 days, reflects a substantial improvement. The current findings demonstrate that a short thermal pre-treatment, when paired with appropriate early-phase feeding, can reliably suppress methane formation during MES start-up without the use of chemical inhibitors. This is further supported by results from a miniaturised version of the used reactor design, where methane was detected within the first 30 days despite BESA addition, highlighting the improved control achieved in the present system (Zegers et al., 2025).

### 3.7 Dissimilatory metabolism under mixotrophic conditions selectively fixes CO_2_ into carboxylic acids

Given that methane accounted for only a minor share of the electron balance, particular attention was given to how electrons were allocated during Stage 1 between biomass formation and the dissimilatory reduction of CO_2_ to carboxylates (Figure 7). When accounting for all electron donors (glucose, fructose, and peptone), it was estimated that in mixotrophic conditions, current contributed approximately 50% of the total charge, representing a 25% increase in total electron input compared to heterotrophic operation. When excluding the contribution of current, the biomass CE in the MT and M reactors approaches the values observed in the HT and H reactors. This suggests that the WLP was not the primary route used for biomass production in the mixotrophic conditions. It is well-established that the degradation of organic substrates increases CO_2_ production, which in turn supports redox balancing in acetogens during the formation of acetyl-CoA (Schuchmann and Müller, 2016; Lechtenfeld et al., 2018). However, it remains unclear where the surplus of acetyl-CoA is used. The current study highlights that it is not primarily used for biomass formation but instead for the production of acetate, butyrate, and hexanoate, indicating its role in energy metabolism. Carboxylate production was higher in reactors M and MT compared to H and HT, with a CE ratio of approximately 3:1 when the highest values are considered. This observation supports the hypothesis that dissimilatory metabolism of CO_2_ is taking place. The results indicate that under the specific experimental and process conditions applied in this study, using defined concentrations of organic substrates in the presence of CO_2_ and an applied current, glucose and fructose are mainly utilised for biosynthetic activity. CO_2_, on the other hand, is reduced via the WLP primarily for energy production through acetate synthesis (Müller et al., 2001; Ragsdale, 2008; Ragsdale and Pierce, 2008; Biegel and Müller, 2010; Schuchmann and Müller, 2014). If a significant portion of the CO_2_ had been used for biomass formation, clearer differences in growth kinetics and overall efficiency would have been expected.

**Figure 8:**
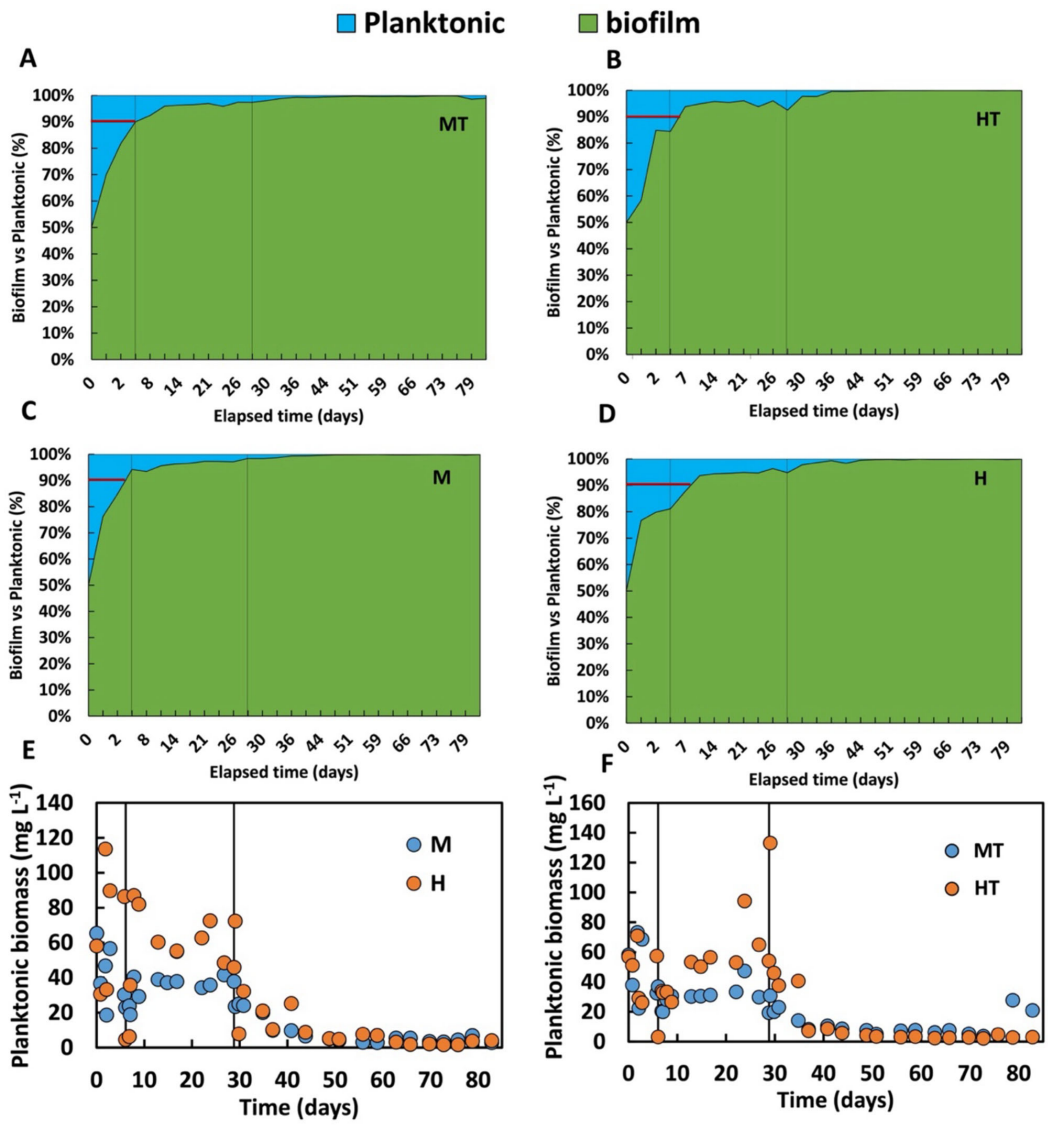
Temporal distribution in percentage of the biomass in the biofilm and in the bulk (planktonic biomass) in the MT **(A)**, HT **(B)**, M **(C)**, H **(D)** reactors, and trend over time of the concentration of planktonic biomass in M and H **(E)**, MT and HT **(F)**. The first solid line indicates the end of the batch period and the beginning of the continuous mode (Stage 1); the second solid line indicates the beginning of the autotrophic phase (Stage 2).

The improved use of organic substrate in anabolic cellular processes may help explain the differences observed in biomass distribution between the biofilm and the bulk phase (Figure 8). Although the M and MT reactors reached approximately 90 percent of total biomass in the biofilm phase within just 5 days, adhesion to the electrode surface was also satisfactory in the H and HT reactors, where similar distribution levels were achieved after around 7 days. However, the biomass distribution profile remained stable throughout Stage 1 in reactors M and MT (Figures 8A–C), whereas in reactors H and HT (Figures 8B–D), the profile appeared more variable, indicating a lower affinity of the biomass for the electrode. This pattern is further supported by Figures 8E, F, where the H and HT reactors show higher concentrations of planktonic biomass, while M and MT demonstrate a stronger tendency for microbial electrode attachment under polarised conditions.

The biofilm-to-planktonic biomass ratios over time indicate greater biofilm stability in reactors M and MT. This suggests that the use of glucose and fructose, possibly linked to dissimilatory CO_2_ metabolism during anabolic processes, may have contributed to the formation of extracellular polymeric substances (EPS) of a different composition. However, a previous study reported that under conditions where both glucose and electric current were present, 50% of the glucose was used for acetate production, with the remainder directed towards biosynthetic pathways (Zaybak et al., 2013). This difference may be related to the lower glucose concentration used in that study (0.36 g L^−1^). In the present work, a higher glucose concentration of 3.5 g L^−1^ was used, which could have shifted the metabolic balance in favour of anabolic processes due to the increased substrate availability.

## 4 Conclusion

This study demonstrates a two-stage approach that promotes the quicker formation of an acetogenic biofilm on the cathode while facilitating suppression of methanogenic activity, without chemical inhibitors. Thermal pre-treatment followed by a mixotrophic start-up phase enriched acetogenic genera such as *Clostridium sensu stricto 12* and *Sporomusa*, reached full electrode colonisation in 55–65 days, and provided a stable platform for subsequent autotrophic operation. The approach initiated acetate, butyrate, and caproate production within the first week, and raised acetate to multi-gram per litre levels by around 3 weeks. Methane remained undetectable for about 40 days and, when detected later, showed low coulombic efficiencies (< 1%). The data suggests a dissimilatory metabolism of CO_2_ via the WLP pathway, with acetyl-CoA channelled to acetate for energy conservation and biomass formation supported by organics.

Relative to heterotrophic start-up, mixotrophy achieved earlier and higher acetate accumulation during start-up. Heat treatment with mixotrophy introduced a short lag phase and contributed to early suppression of methane CE. However, *Methanobrevibacter* was detectable in the final biofilm of this combined approach and chain elongation was less sustained than in the mixotrophic approach alone, consistent with the possibility that heat treatment also affected chain-elongating genera. Taken together, these outcomes support mixotrophic start-up as a practical route to rapid, stable MES operation without reliance on inhibitors. Further optimisation is needed to shorten the start-up phase more and extend the period of methanogenesis suppression while preserving chain-elongation potential and long-term productivity. Additionally, work is needed to determine whether the low methane concentrations reflect suppressed methanogenesis or simply low-rate maintenance, and to define operating windows that sustain acetogenesis while inhibiting methanogenesis.

## Data Availability

The original contributions presented in the study are included in the article/Supplementary material. V3-V4 16S rRNA gene amplicon sequencing reads have been deposited in the NCBI Sequence Read Archive (SRA) under BioProject PRJNA1344572. Further inquiries can be directed to the corresponding author.
