# Supplementary material for: A two-stage strategy for methanogenesis suppression and rapid acetogenic biofilm formation in microbial electrosynthesis
Source: Front Microbiol. 2025 Oct 31;16:1655259. doi: 10.3389/fmicb.2025.1655259 (PMC12616864; doi:10.3389/fmicb.2025.1655259)
Supplement: Supplementary file 1 [file Data_Sheet_1.pdf]

A Two-Stage Strategy for Methanogenesis Suppression and Rapid Acetogenic Biofilm Formation in Microbial Electrosynthesis

Supplementary Information

Jacopo Ferretti[1†], Marika A.J. Zegers[2,3†], M. Zeppilli[1], Ludovic Jourdin[2,3*]

[1]Department of Chemistry, University of Rome Sapienza, Piazzale Aldo Moro 5, 00185 Roma, Italy

[2]Department of Biotechnology, Delft University of Technology, van der Maasweg 9, 2629 HZ Delft, the Netherlands

[3]e-Refinery Institute, Delft University of Technology, Leeghwaterstraat 39, 2628 CB Delft, the Netherlands

[†]These authors contributed equally to this work and share first authorship

*Correspondence:
Corresponding Author
L.Jourdin@tudelft.nl

1. Reactor Setup

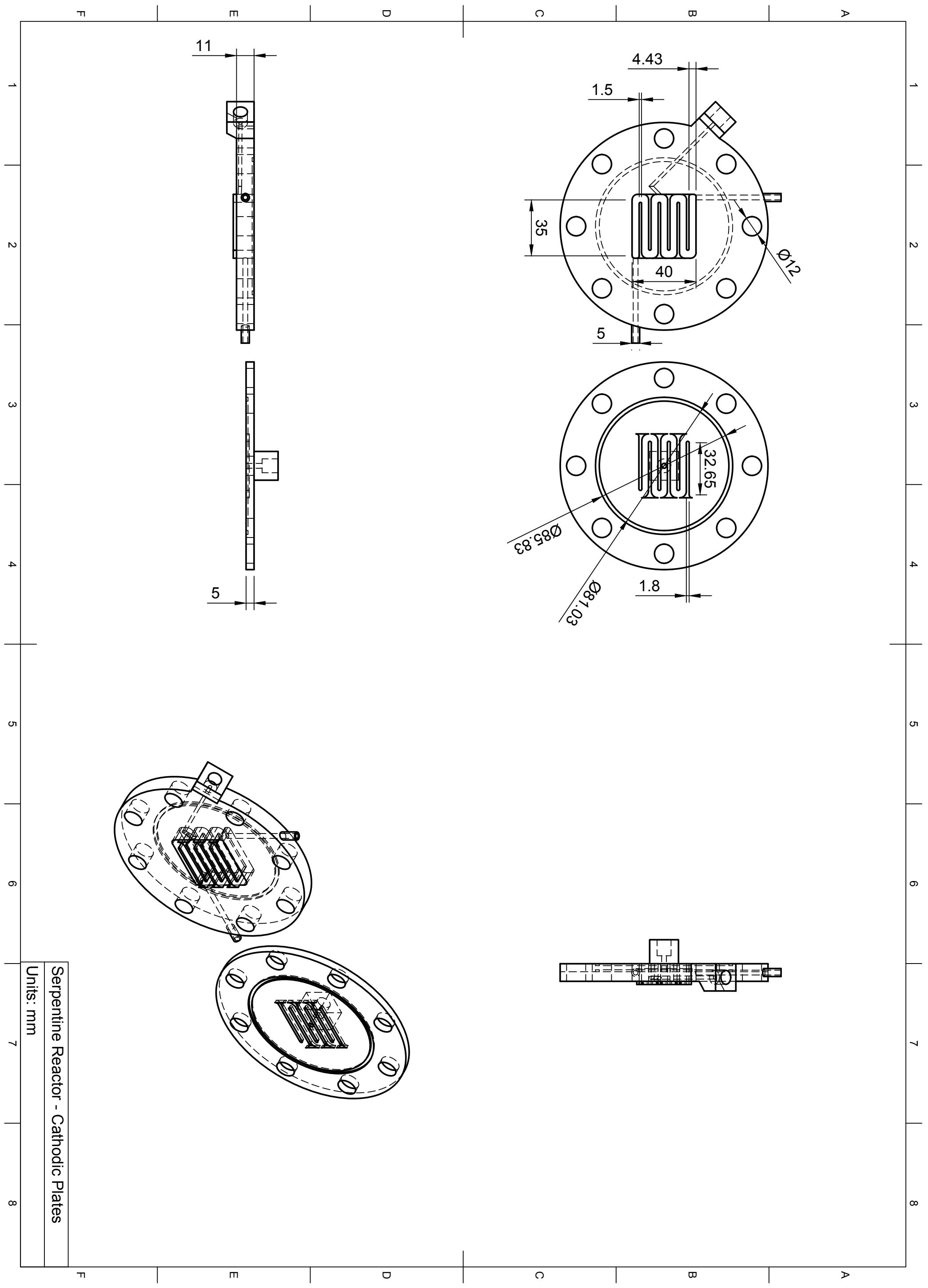

Figure S1: Sketch of the cathodic plates of the serpentine directed-flow-through bioelectrochemical reactor and corresponding dimensions (mm).

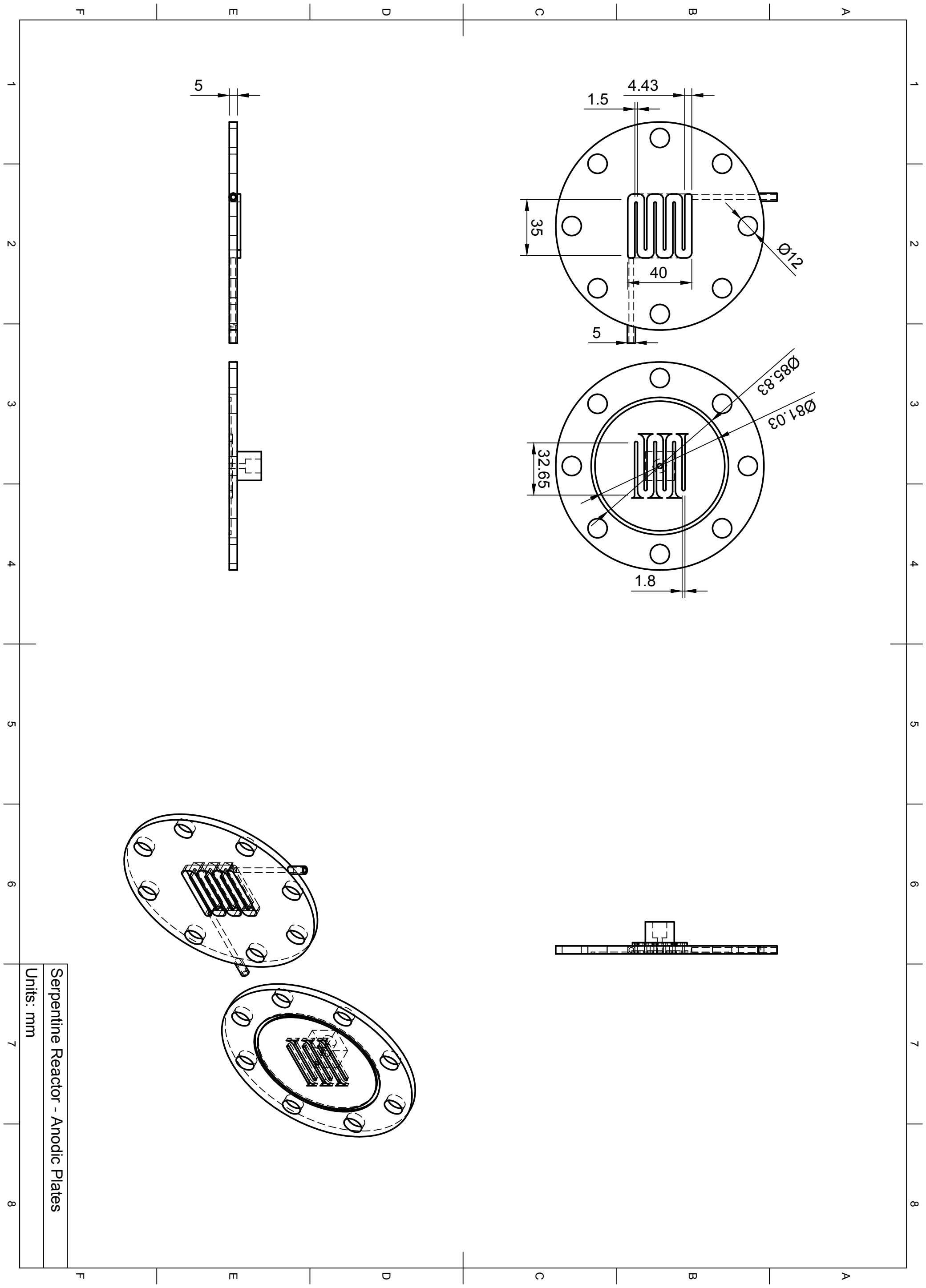

Figure S2: Sketch of the anodic plates of the serpentine directed-flow-through bioelectrochemical reactor and corresponding dimensions (mm).

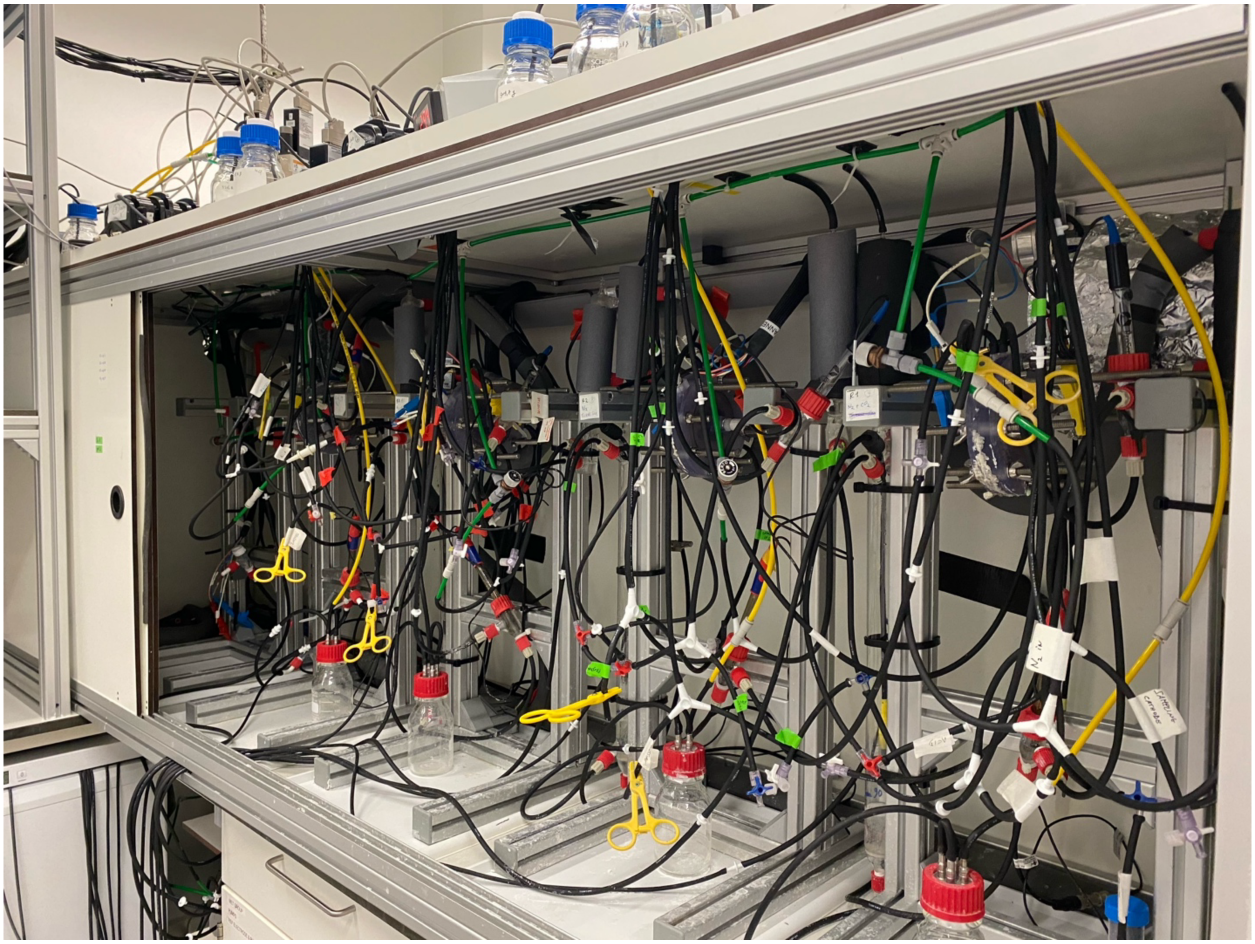

Figure S3: The four serpentine directed-flow-through bioelectrochemical reactor setups used in this work.

2. Cell Voltage–Time Series

Notes on variability in cell voltage at fixed current. Small differences in cathode assembly and material properties can result in differences in electrochemical performance, even when reactor geometry and operating conditions are the same. In particular, variation in the carbon felt–current collector contact (e.g., graphite adhesive coverage/thickness and local compression) impacts contact resistance, and inherent felt microstructure/porosity heterogeneity alters current distribution (Zegers et al., 2025). Accordingly, the voltage profiles shown here should be interpreted with this assembly/material variability in mind.

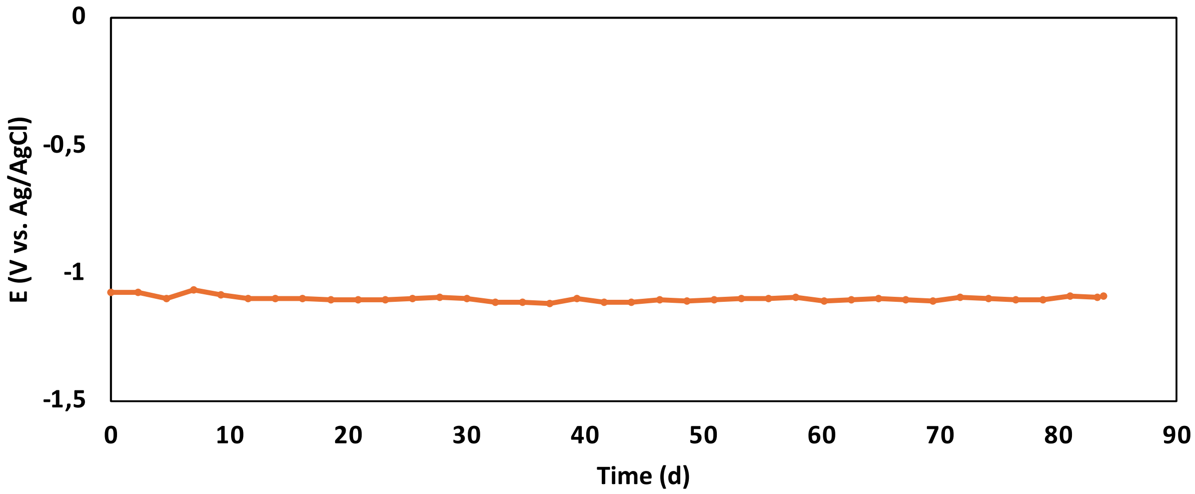

Figure S4: Cathode potential (vs. Ag/AgCl, 3 M KCl) over time for reactor M at an applied current of -63 mA (-5.04 mA $cm^{-2}{}_{PSA}$).

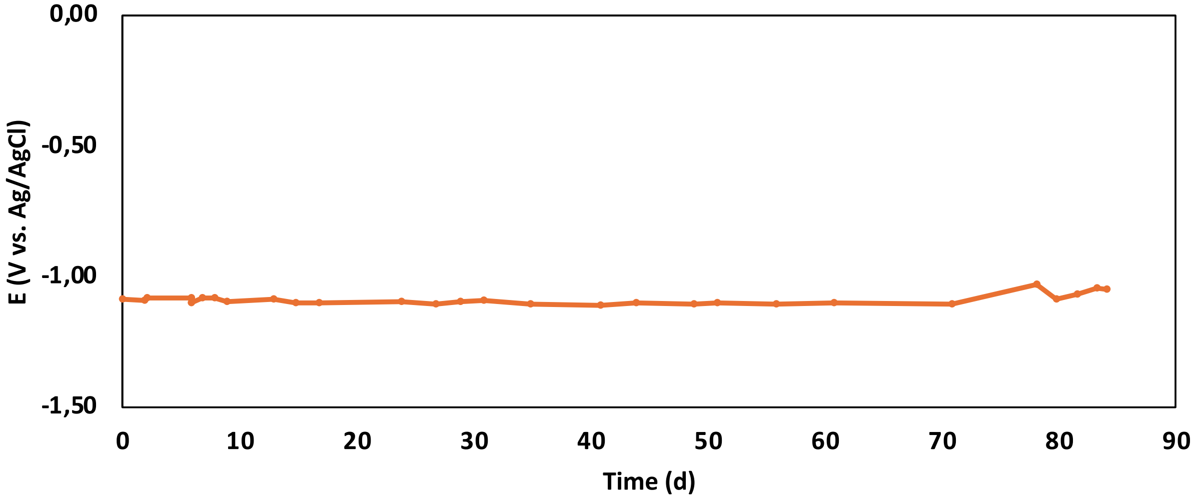

Figure S5: Cathode potential (vs. Ag/AgCl, 3 M KCl) over time for reactor MT at an applied current of -63 mA (-5.04 mA $cm^{-2}{}_{PSA}$).

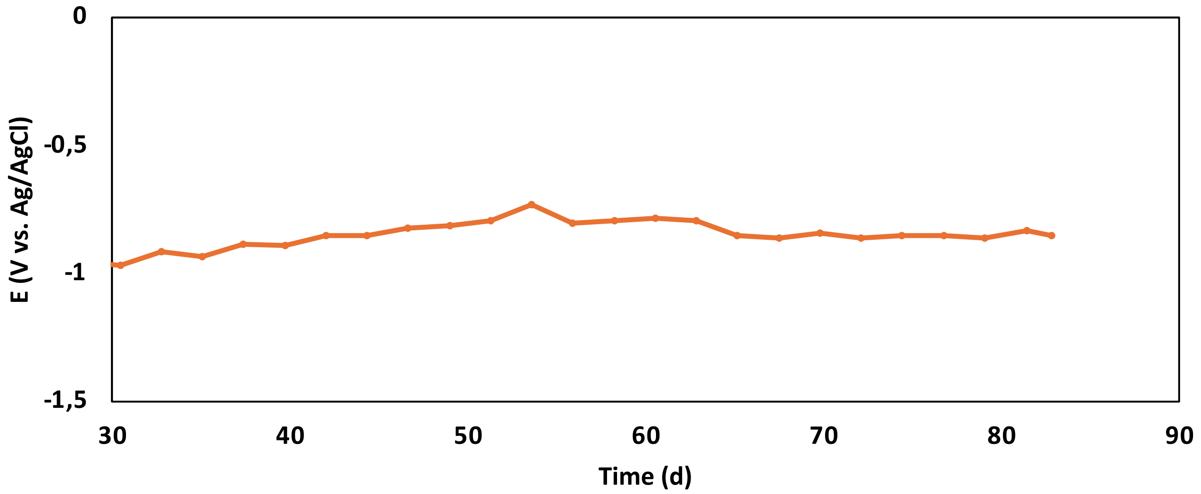

Figure S6: Cathode potential (vs. Ag/AgCl, 3 M KCl) over time for reactor H at an applied current of -63 mA (-5.04 mA $cm^{-2}{}_{PSA}$).

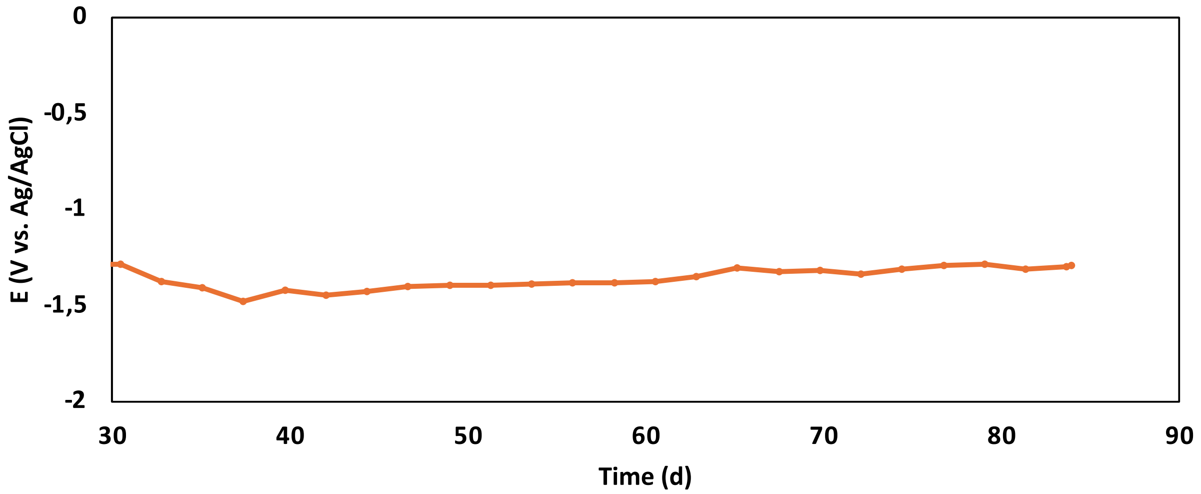

Figure S7: Cathode potential (vs. Ag/AgCl, 3 M KCl) over time for reactor HT at an applied current of -63 mA (-5.04 mA $cm^{-2}{}_{PSA}$).

3. Reactor Recirculation Loops

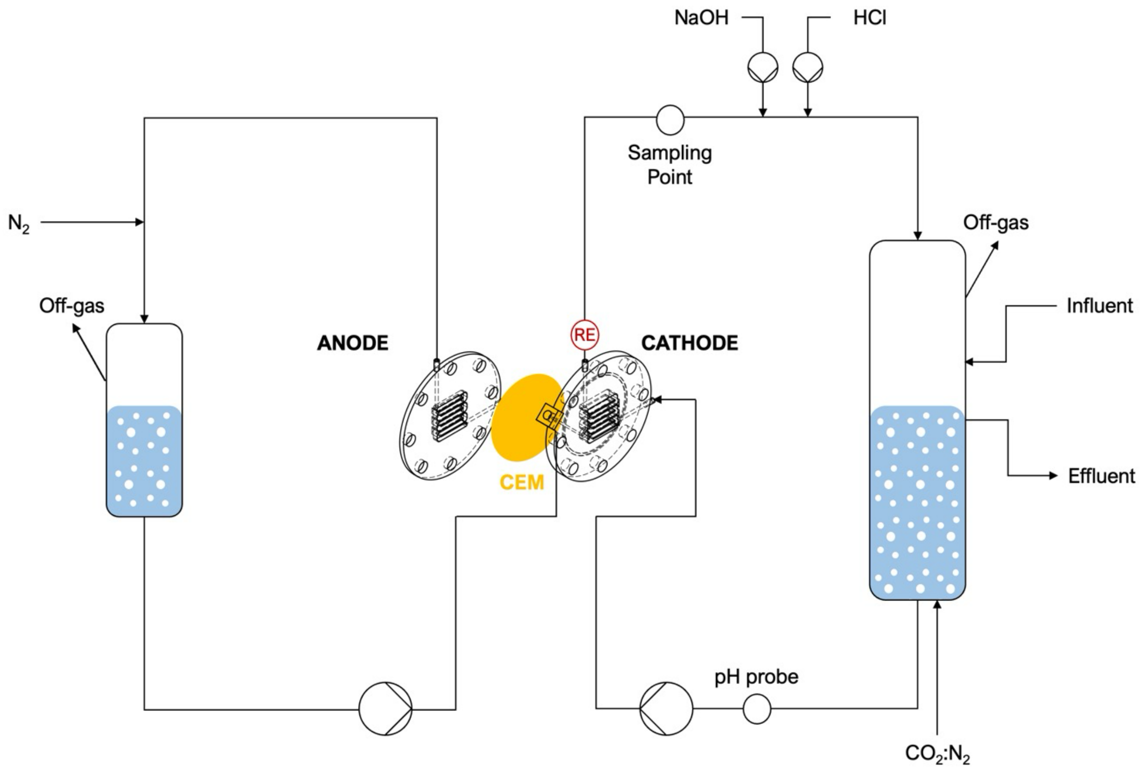

Figure S8: Schematic of the complete serpentine directed-flow-through bioelectrochemical reactor. RE: reference electrode.

4. Mass Balances

The mass balance for each reactor's cathode compartment was defined as:

$$\frac{dn_i}{dt} = F_{in}c_{i,in} - F_{out}c_{i,out} + r_i V_C$$

Where n_i is the mole amount of compound *i*, *t* is time (d), *F* is the flow rate (L d^{-1}), $c_{i,in}$ is the ingoing concentration (0 mol_i L^{-1} for products in this study), $c_{i,out}$ is the outgoing concentration, r_i is the volume-specific production rate of *i* (mol_i L^{-1} d^{-1}) and V_C is the total catholyte volume (L). The titrant flow, F_{pH} was much smaller than F_{in}. Therefore, we disregarded it in this study, and we assumed $F_{in} = F_{out} = F$.

Coulombic efficiency (CE%), or electron recovery, is defined as the total amount of electric charge retrieved in the products of interest (organics and biomass), $Q_{products}$ (coulomb), divided by the total charge input Q_T (coulomb). Here, Q_T is the sum of the electrical charge supplied and the electron equivalents from consumed organics (glucose, fructose, peptones) during Stage 1. Under autotrophic Stage 2, Q_T is the electrical charge only.

$$CE\% = \frac{Q_{products}}{Q_T} \cdot 100\%$$

References

Zegers, M. A. J., Augustijn, E., Jongbloed, G., and Jourdin, L. (2025). Novel miniaturised microbial electrosynthesis reactor: A study on replicability. *Chemical Engineering Journal* 516, 163881. doi: 10.1016/j.cej.2025.163881
